# Zeta-Fe_2_O_3_ – A new stable polymorph in iron(III) oxide family

**DOI:** 10.1038/srep15091

**Published:** 2015-10-15

**Authors:** Jiří Tuček, Libor Machala, Shigeaki Ono, Asuka Namai, Marie Yoshikiyo, Kenta Imoto, Hiroko Tokoro, Shin-ichi Ohkoshi, Radek Zbořil

**Affiliations:** 1 Regional Centre of Advanced Technologies and Materials, Departments of Physical Chemistry and Experimental Physics, Faculty of Science, Palacky University, Slechtitelu 27, 783 71 Olomouc, Czech Republic; 2 Research and Development Center for Ocean Drilling Science, Japan Agency for Marine-Earth Science and Technology, 2-15 Natsushima-cho, Yokosuka-shi, Kanagawa 237-0061, Japan; 3 Department of Chemistry, School of Science, The University of Tokyo, 7-3-1 Hongo, Bunkyo-ku, Tokyo 113-0033, Japan

## Abstract

Iron(III) oxide shows a polymorphism, characteristic of existence of phases with the same chemical composition but distinct crystal structures and, hence, physical properties. Four crystalline phases of iron(III) oxide have previously been identified: α-Fe_2_O_3_ (hematite), β-Fe_2_O_3_, γ-Fe_2_O_3_ (maghemite), and ε-Fe_2_O_3_. All four iron(III) oxide phases easily undergo various phase transformations in response to heating or pressure treatment, usually forming hexagonal α-Fe_2_O_3_, which is the most thermodynamically stable Fe_2_O_3_ polymorph under ambient conditions. Here, from synchrotron X-ray diffraction experiments, we report the formation of a new iron(III) oxide polymorph that we have termed ζ-Fe_2_O_3_ and which evolved during pressure treatment of cubic β-Fe_2_O_3_ (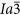 space group) at pressures above 30 GPa. Importantly, ζ-Fe_2_O_3_ is maintained after pressure release and represents the first monoclinic Fe_2_O_3_ polymorph (*I*2/*a* space group) that is stable at atmospheric pressure and room temperature. ζ-Fe_2_O_3_ behaves as an antiferromagnet with a Néel transition temperature of ~69 K. The complex mechanism of pressure-induced transformation of β-Fe_2_O_3_, involving also the formation of Rh_2_O_3_-II-type Fe_2_O_3_ and post-perovskite-Fe_2_O_3_ structure, is suggested and discussed with respect to a bimodal size distribution of precursor nanoparticles.

Iron(III) oxide is a polymorphic compound, i.e., it can exist in two or more solid phases that are isochemical but have distinct crystal structures and thus different physical properties. Under ambient conditions, four different crystalline polymorphs of iron(III) oxide have been discovered and characterized in details^1^,^2^,^3^,^4^,^5^: (i) α-Fe_2_O_3_, mineralogically known as hematite, which has a rhombohedrally centred hexagonal crystal structure (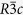 space group with *a* = 5.034 Å and *c* = 13.752 Å); (ii) β-Fe_2_O_3_, which has a cubic body-centred crystal structure of bixbyite type (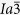 space group with *a* = 9.393 Å); (iii) γ-Fe_2_O_3_, mineralogically known as maghemite, which has a cubic crystal structure of inverse spinel type (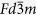 space group with *a* = 8.351 Å and vacancies disordered over the octahedral cation sites in the crystal lattice); and (iv) ε-Fe_2_O_3_, which has an orthorhombic crystal structure (*Pna*2_1_ space group with *a* = 5.072 Å, *b* = 8.736 Å, and *c* = 9.418 Å). While α-Fe_2_O_3_ and γ-Fe_2_O_3_ are naturally abundant and can be prepared in diverse morphological forms with different sizes by various optimized synthetic routes, β-Fe_2_O_3_ and ε-Fe_2_O_3_ are rarely observed in nature and their stability is dependent on the nanodimensional character of their particles^1^,^2^,^3^,^4^,^5^,^6^.

Due to their different physical properties, which arise from the differences in their crystal structures, all of the iron(III) oxide polymorphs have found applications in nanotechnology or show considerable promise in such applications. For instance, thin nanocrystalline films of α-Fe_2_O_3_ serve as very efficient electrodes in the photo-assisted electrolysis of water for hydrogen production in solar cells^7^,^8^,^9^ and α -Fe_2_O_3_ nanoparticles are effective catalysts for various processes of heterogeneous catalysis^10^,^11^,^12^. β-Fe_2_O_3_ was recently used as a chloroform sensor^13^ and identified as a suitable candidate for the preparation of anodes in lithium-ion batteries^14^. Ferrimagnetic and superparamagnetic nanoparticles of γ-Fe_2_O_3_, the most widely used iron(III) oxide polymorph, have been identified as a possible functional medium for magnetocaloric refrigeration^15^. They also have diverse biomedical applications because they are biocompatible and biodegradable while exhibiting useful magnetic properties; they have been used as MRI contrast agents, carriers for targeted drug delivery, heating units in magnetically-induced cancer therapy (i.e., hyperthermia), and sensors of various biologically important molecules^16^,^17^,^18^,^19^. The most recently identified iron(III) oxide polymorph, ε-Fe_2_O_3_, shows the highest coercivity among all known metal oxides (20–22.5 kOe)^4^,^6^ and could therefore be used as a magnetic recording material for high-density recording media. In addition, its magnetoelectric properties predestine that it could be useful in the production of multiple-state-memory elements^4^,^20^. Finally, it exhibits ferromagnetic resonance in millimetre wave region, giving it potential applications in devices for suppressing electromagnetic interference and stabilizing electromagnetic transmittance^4^,^21^,^22^,^23^.

Due to the diverse potential applications of all four known iron(III) oxide polymorphs, thermally induced phase transformations of the less thermodynamically stable polymorphs (β-, γ-, and ε-Fe_2_O_3_) have been studied extensively. The results of these transformations depend on the intrinsic properties of the starting phase (polymorph structure, particle size, particle morphology, surface coating, particle aggregation, incorporation of particles within a matrix) and the nature of the applied treatment. In general, such transformations ultimately yield α-Fe_2_O_3_ as the final product and are frequently accompanied with the particle growth^5^,^24^. The two rare iron(III) oxide polymorphs, β- and ε-Fe_2_O_3_, undergo direct thermal transitions to α-Fe_2_O_3_^5^. However, hollow β-Fe_2_O_3_ nanoparticles can be transformed into γ-Fe_2_O_3_^5^,^25^, demonstrating that the morphology of the starting material can significantly affect the transformation process and permit the evolution of unexpected intermediate iron(III) polymorphs. In the case of γ-Fe_2_O_3_, both direct transformations into α-Fe_2_O_3_ and two-step transformations via ε-Fe_2_O_3_ have been observed, depending on the initial particle size and degree of interparticle interactions (i.e., aggregation, the extent to which the particles are spatially confined)^3^,^4^,^5^.

Although thermally induced transformations of Fe_2_O_3_ polymorphs have been described at length in the literature, pressure-induced transitions have only been investigated for the most common polymorphs, α-Fe_2_O_3_ and γ-Fe_2_O_3_^5^. Several studies aiming to simulate the geophysical conditions in the Earth’s lower mantle have examined the behaviour of α-Fe_2_O_3_ under ultra-high pressures^26^,^27^,^28^,^29^,^30^,^31^,^32^,^33^. For instance, Ito *et al.*^26^ observed consecutive transitions of α-Fe_2_O_3_ to the Rh_2_O_3_-II-type Fe_2_O_3_ structure (*Pbcn* space group) and then to an orthorhombic structure (with *a* = 6.883 Å, *b* = 9.993 Å, *c* = 4.665 Å, and *V* = 320.9 Å^3^) as the applied pressure was increased from atmospheric levels to 58 GPa at 1400 K. Ono *et al.*^27^ observed that the application of a pressure of 30 GPa with laser heating at 2000 K resulted in the formation of Fe_2_O_3_ with a perovskite-type structure (*Pbnm* space group). When the pressure was increased to 70 GPa and the temperature to 2500 K, other perovskite-like structures (so-called post-perovskites) with both orthorhombic (with *a* = 2.639 Å, *b* = 6.386 Å, *c* = 8.544 Å, and *V* = 144.0 Å^3^) and monoclinic (*a* = 5.282 Å, *b* = 6.385 Å, *c* = 4.471 Å, β = 107.22°, and *V* = 144.0 Å^3^) symmetry were identified. A further increase in pressure (to 96 GPa) and temperature (to 2800 K) resulted in the conversion of the perovskite structure to a CaIrO_3_-type structure with an orthorhombic symmetry (*Cmcm* space group)^28^. Recently, Bykova *et al.*^32^ reported the pressure-induced transformation of α-Fe_2_O_3_ single crystals into a cryolite double-perovskite-type phase with a monoclinic unit cell (*P*2_1_/*n* space group) at about 54 GPa; the pressure-induced transition is accompanied by a large compression in the unit cell not previously observed for the α-Fe_2_O_3_/perovskite/post-perovskite pathway. In addition to these changes in the crystal structure and unit cell volume of α-Fe_2_O_3_, mild increases in the applied pressure have been observed to affect some of its other physical properties. Among other things, pressure treatment has been reported to increase its Morin transition temperature^34^, both to increase and decrease its electrical conductivity^35^,^36^,^37^, induce a high-spin to low-spin transition (i.e., a 5/2-to-1/2 spin crossover)^29^, and cause the disappearance of a magnetically-ordered state^35^. Most importantly, all previously reported high-pressure transformations of α -Fe_2_O_3_ (whether they occur at room temperature or under heating) were found to be reversible, i.e., the material recovered after the pressure was released had the original hexagonal crystal structure of α-Fe_2_O_3_ with almost unmodified physicochemical properties.

Conversely, high-pressure treatments of γ-Fe_2_O_3_ phase typically cause its irreversible transformation to α-Fe_2_O_3_, which is followed by the evolution of perovskite and post-perovskite structures identical to those formed during pressure treatment of α-Fe_2_O_3_^38^,^39^,^40^,^41^,^42^,^43^,^44^. The pressure required to initiate the γ-Fe_2_O_3_-to-α-Fe_2_O_3_ phase transformation ranges from ~10 to ~37 GPa and appears to be highly dependent on the particle size of the transformed γ-Fe_2_O_3_ phase. For instance, Clark *et al.*^38^ confirmed that the transition pressure increases as the size of the γ-Fe_2_O_3_ nanocrystals decreases: the pressures required to transform nanocrystals with dimensions of 7, 5, and 3 nm were 27 GPa, 34 GPa, and 37 GPa, respectively. This trend was attributed to the higher surface energy of smaller nanocrystals.

There are no previous reports on the high-pressure transformations of the rare β-Fe_2_O_3_ and ε-Fe_2_O_3_ phases. Here, we describe the high-pressure transformations of rare β-Fe_2_O_3_ for the first time. β-Fe_2_O_3_ has a cubic crystal structure containing two non-equivalent octahedral cation sites, which have distinct symmetries and are referred to as b-sites and d-sites. They are filled with Fe^3+^ ions in a high-spin state (*S* = 5/2). On lowering the temperature, β-Fe_2_O_3_ passes from a paramagnetic to a magnetically ordered regime, adopting an antiferromagnetic state below ~110 K (the Néel temperature). The X-ray synchrotron data presented in this study show that when β-Fe_2_O_3_ is exposed to pressures above 30 GPa, a new iron(III) oxide polymorph designated zeta-Fe_2_O_3_ (ζ-Fe_2_O_3_) is formed. Remarkably, this new polymorph remains stable at room temperature, even after the pressure is released. It has a monoclinic crystal structure, belonging to the *I*2/*a* space group. Magnetization measurements indicate that it behaves as an antiferromagnet at temperatures below ~69 K. Its stability is explained due to the high surface energy it gains by being formed from smaller β-Fe_2_O_3_ nanoparticles, and favourable changes in its chemical potential that occur during pressure treatment.

## Results and Discussion

**Figure 1:**
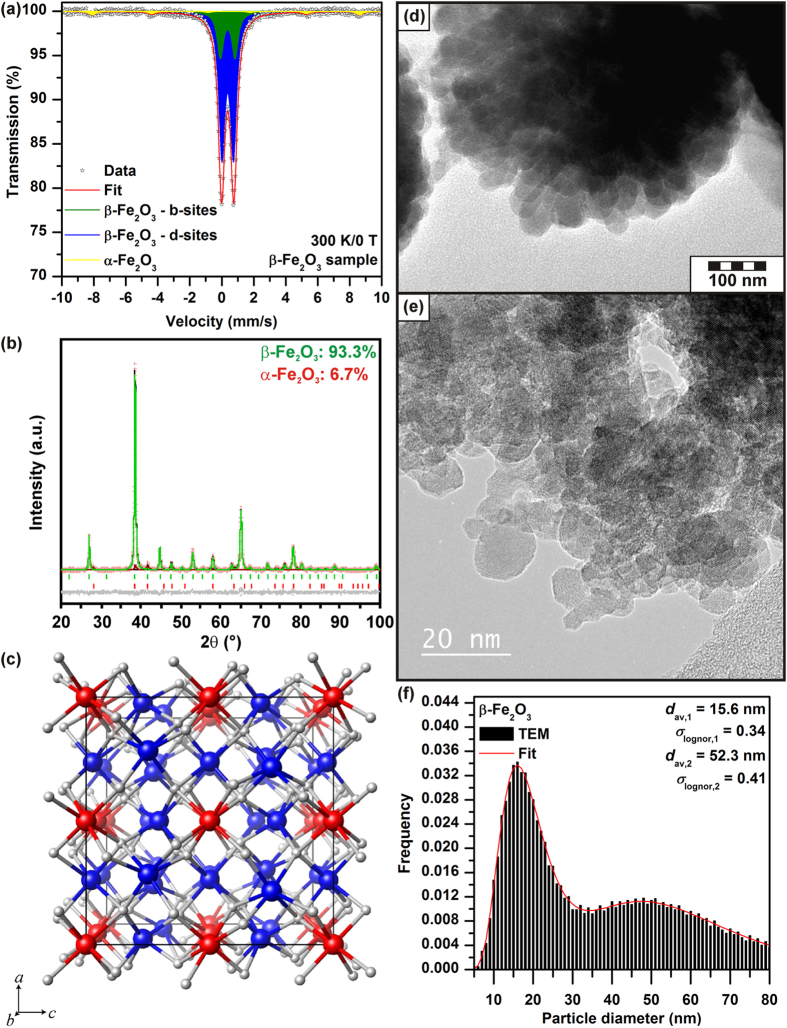
Characterization of the starting b-Fe_2_O_3_ sample. (**a**) Room-temperature ^57^Fe Mössbauer spectrum and (**b**) conventional XRD pattern of β-Fe_2_O_3_ sample before its treatment under high pressures. (**c**) Crystal structure of β-Fe_2_O_3_ (cubic, 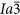 space group) projected along the *b*-axis. Red, blue, and gray balls represent the octahedral Fe b-sites, octahedral Fe d-sites, and oxygen sites, respectively. (**d**,**e**) TEM and HRTEM image showing fraction of smaller and larger β-Fe_2_O_3_ nanoparticles and (**f**) particle size distribution derived from TEM/HRTEM images, where bars correspond to experimentally observed nanoparticle sizes and red curve represents the best theoretical fit employing two lognormal distribution curves.

Before its pressure treatment, the purity and structural features of the synthesized β-Fe_2_O_3_ sample were checked using conventional X-ray powder diffraction (XRD) and ^57^Fe Mössbauer spectroscopy. The room-temperature ^57^Fe Mössbauer spectrum of the β-Fe_2_O_3_ sample is well deconvoluted into 3 spectral components – two dominant doublets whose isomer shift and quadrupole splitting values are characteristic of the b-sites and d-sites in the β-Fe_2_O_3_ crystal lattice (with an ideal spectral ratio of 1:3 in accordance with the complete occupation of individual crystallographically non-equivalent cation positions by Fe^3+^) and a minor sextet typical of α-Fe_2_O_3_ admixture (see Fig. 1a and Table 1). Based on the spectral areas of these components, the level of α-Fe_2_O_3_ admixture was 7 wt.%, a conclusion supported by the material’s XRD pattern (see Fig. 1b). The crystal structure of the β-Fe_2_O_3_ sample, derived by the Rietveld analysis of its XRD pattern, is shown in Fig. 1c. It has a cubic crystal structure within the 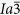 space group, lattice parameters of *a* = *b* = *c* = 9.404 Å, and a unit volume of *V* = 831.8 Å^3^.

High-resolution transmission electron microscopy (HRTEM/TEM) images of the prepared β-Fe_2_O_3_ sample (see Fig. 1d,e) indicate that it contains nanoparticles of two distinct size classes (hereafter referred to as smaller and larger nanoparticle assemblies). It turns out that the two size fractions are well described (employing the *χ*^2^-test performed on a statistical level of confidence of 99%) in terms of lognormal distribution curves with average particle sizes of 15.6 and 52.3 nm and lognormal standard deviations of 0.34 and 0.41, respectively (see Fig. 1f). Assuming that the density of β-Fe_2_O_3_ is irrespective of particle size within the sample and nanoparticles are more or less spherical (see Fig. 1d,e), from the frequency vs. size distribution, the smaller and larger nanoparticle assemblies within the β-Fe_2_O_3_ sample account for 36.9(5) and 63.1(5) wt.%, respectively. It is believed that the formation of the two particle fractions is due to the solid-state reaction outlined in Eq. (1)^45^. This thermally-induced process occurs at a temperature of 400 °C, which is well below the decomposition temperature of iron(III) sulphate and the transition temperature for the transformation of β-Fe_2_O_3_ into α-Fe_2_O_3_ (~500 °C)^2^,^5^. However, the double sulphates formed by the reaction outlined in Eq. (1) may also be transformed into β-Fe_2_O_3_. As such, there are two parallel processes of β-Fe_2_O_3_ formation – the primary reaction and the subsequent decomposition of the double sulphates – leading to the formation of the two particle size fractions observed after complete removal of all the sulphate-based by-products via dissolution in water.
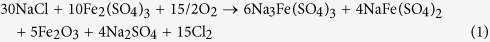


**Figure 2:**
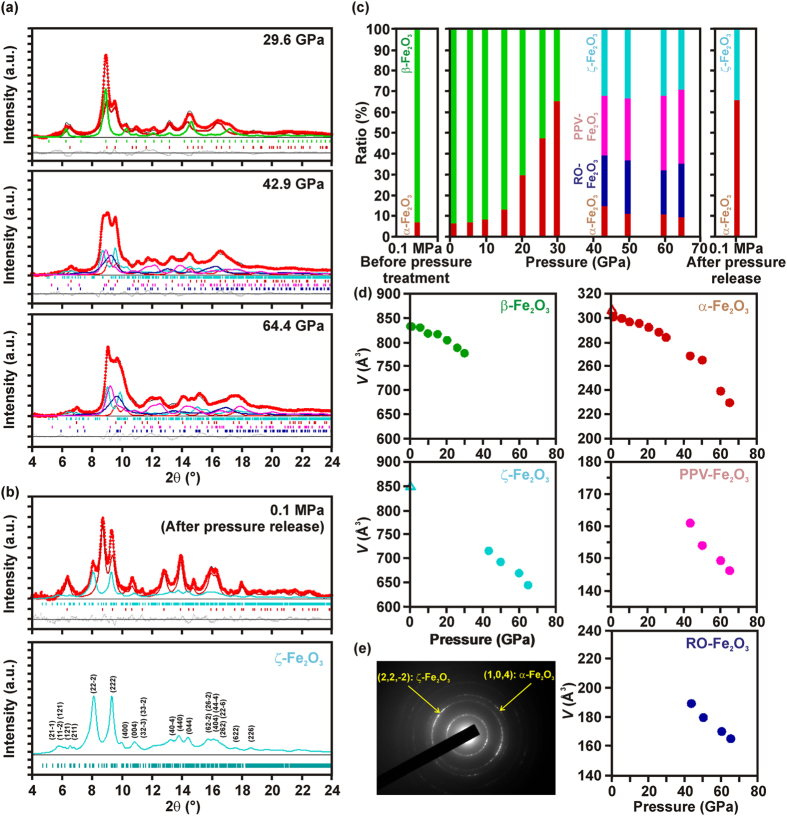
Synchrotron radiation XRD data for the treated b-Fe_2_O_3_ phase. Synchrotron radiation XRD patterns with Rietveld analysis acquired (**a**) at various elevated pressures and (**b**) at 0.1 MPa (atmospheric pressure) after pressure release. Red dots, black lines, and gray dots indicate the observed patterns, fitted patterns, and the differences between them, respectively. Fitted patterns for each phase are shown with green lines (β-Fe_2_O_3_), brown lines (α-Fe_2_O_3_), navy blue lines (RO-Fe_2_O_3_), pink lines (PPV-Fe_2_O_3_), and light blue lines (ζ-Fe_2_O_3_). The tick marks indicate the calculated positions of the Bragg reflections for each phase. (**d**) Cell volume vs. pressure plots for β-Fe_2_O_3_ (green), ζ-Fe_2_O_3_ (light blue), α-Fe_2_O_3_ (brown), RO-Fe_2_O_3_ (navy blue), and PPV-Fe_2_O_3_ (pink). The open triangles show the cell volume of ζ-Fe_2_O_3_ (light blue) and α-Fe_2_O_3_ (brown) after pressure release. (**e**) SAED pattern of the sample after pressure release with identification of the most intense diffraction plane belonging to α-Fe_2_O_3_ and ζ-Fe_2_O_3_ (no diffraction corresponds to β-Fe_2_O_3_).

The effect of pressure treatment on the crystal structure of β-Fe_2_O_3_ was investigated using high-pressure synchrotron radiation XRD measurements. Representative high-pressure synchrotron XRD spectra are shown in Fig. 2a and the detailed Rietveld analyses of all the measured synchrotron radiation XRD patterns (including the values of the *R*_wp_-factor) are depicted in Supplementary Figures S1–S7 in the Supplementary Material. At pressures of up to 10 GPa, the sample consist of β-Fe_2_O_3_ and α-Fe_2_O_3_ in approximately the same ratio as in the original sample (93/7 wt.%). This reflects the pressure stability of β-Fe_2_O_3_ up to 10 GPa. The crystal structure of β-Fe_2_O_3_ was determined, and that of the increasing α-Fe_2_O_3_ phase was refined sequentially between 10 and 30 GPa. Some of the β-Fe_2_O_3_ nanoparticles undergo polymorphous transformation to α-Fe_2_O_3_ but no other iron(III) polymorphs are observed (see Fig. 2a,c). At 29.6 GPa, the fractions of β-Fe_2_O_3_ and α-Fe_2_O_3_ were 35.1(2)% and 64.9(2)%, respectively (see Fig. 2c). Given the relative volume (mass) ratio of the two β-Fe_2_O_3_ particle size fractions in the starting material (as determined from TEM/HRTEM analysis, see above), this implies that the smaller nanoparticle assembly remains untransformed but the larger nanoparticle assembly readily converts into α-Fe_2_O_3_. One might expect this trend for the conversion of β-Fe_2_O_3_ into α-Fe_2_O_3_ to continue as the pressure increases further. However, there was a dramatic shift in the mechanism of the pressure-induced transformation when the applied pressure was raised above 30 GPa, with both the α-Fe_2_O_3_ and β-Fe_2_O_3_ phases undergoing new structural transformations. Specifically, α-Fe_2_O_3_ was converted into Rh_2_O_3_-II-type Fe_2_O_3_ (RO-Fe_2_O_3_, orthorhombic, *Pbcn* space group) and post-perovskite Fe_2_O_3_ (PPV-Fe_2_O_3_, orthorhombic, *Cmcm* space group) structures (see Fig. 2a,c). Both these phases have previously been observed during high-pressure treatment of α-Fe_2_O_3_. The simultaneous formation of perovskite and post-perovskite structures can be understood by considering the particle size (and, hence, volume) distribution within the larger nanoparticle assembly. The applied pressure forces the crystal structure of α-Fe_2_O_3_ to change but the magnitude of the change is highly dependent on the level of strain inside the nanoparticles, which tends to resist structural alteration. This strain in turn varies considerably with particle size. The transformation process that will occur is that associated with the lowest overall Gibbs free energy, which is influenced by the strain and associated stress. In addition to these (post)-perovskite structures, some α-Fe_2_O_3_ nanoparticles remain untransformed because their size (and, hence, strain) is such that they can resist the effect of the applied pressure.

Surprisingly, β-Fe_2_O_3_ was found to transform into a completely new crystal structure following the Rietveld refinement of the synchrotron radiation XRD patterns recorded at pressures above 30 GPa (see Fig. 2a,c). The analyses were carried out adopting the following scenario. At high pressures (42.9–64.4 GPa), α-Fe_2_O_3_ transforms into RO-Fe_2_O_3_ and PPV-Fe_2_O_3_ with consistent transition pressures compared with the previous reports^26^,^27^,^28^,^29^,^30^,^31^,^32^,^33^. On the other hand, β-Fe_2_O_3_ transits to a different new phase. By indexing these new peaks, we searched for its space group and found a suitable candidate of the crystal structure. The new peak pattern was found to belong to a single phase with a monoclinic crystal structure in the *I*2/*a* space group with the lattice parameters similar to β-Fe_2_O_3_. We know that the structure of this new phase is caused by lowering the symmetry from cubic β-Fe_2_O_3_ to a monoclinic structure. We designated this new phase as ζ-Fe_2_O_3_. ζ-Fe_2_O_3_ has thus a monoclinic crystal structure with a space group of *I*2/*a* and lattice constants of *a* = 9.17 Å, *b* = 9.30 Å, *c* = 8.50 Å, angle *β* = 97.6°, and a unit volume of *V* = 718.4 Å^3^ at 42.9 GPa. While the new iron(III) oxide phase has several structural features that resemble those of its precursor (cubic β-Fe_2_O_3_), it also exhibits some unusual pressure-induced changes in its crystal lattice. In particular, the octahedral Fe b-site splits into two non-equivalent Fe sites (Fe1 and Fe4 with a 1:1 ratio), the octahedral Fe d-site splits into four non-equivalent Fe sites (Fe2, Fe3, Fe5 and Fe6 with a 1:1:2:2 ratio), and the *β* angle between the *a*- and *c*-axis increases to 98° compared to 90° for β-Fe_2_O_3_. At pressures of 42.9–64.4 GPa, the relative abundances of the four phases, i.e., α-Fe_2_O_3_, RO-Fe_2_O_3_, PPV-Fe_2_O_3_, and ζ-Fe_2_O_3_ were almost constant (see Fig. 2c). This indicates that the ζ-Fe_2_O_3_ polymorph is much more stable at high pressures than γ-Fe_2_O_3_, which is transformed into α-Fe_2_O_3_ (and then into perovskite or post-perovskite phases) once the pressure exceeds ~37 GPa^38^. Moreover, the pressure dependence of each phase was found to be monotonous, and the cell volume (see Fig. 2d) and cell parameters (see Supplementary Figure S8) gradually changed as the pressure increased. When examining the XRD data, it should be stressed that the XRD patterns of β-Fe_2_O_3_ and ζ-Fe_2_O_3_ are totally distinct with different number of peaks. This implies that the symmetry of the crystal structure became lower (cubic → monoclinic) and the XRD pattern of ζ-Fe_2_O_3_ cannot be reproduced by modifying the pattern of β-Fe_2_O_3_ considering the effects of strains and defects (peak shift, peak broadening, changing the peak intensities, etc.).

**Figure 3:**
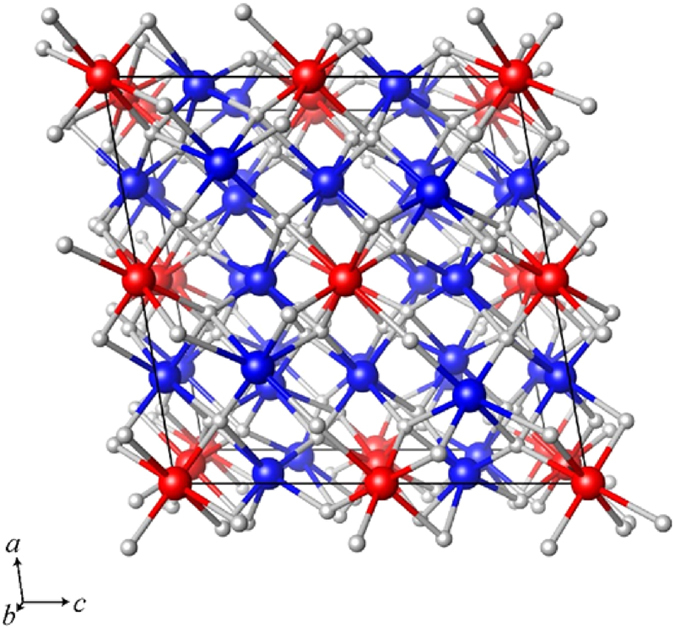
Crystal structure of z-Fe_2_O_3_ (monoclinic, *I*2/*a* space group) after pressure release, projected along the *b*-axis. Red, blue, and gray balls represent Fe sites split from d-sites in β-Fe_2_O_3_ (designated Fe1, Fe4), Fe sites split from b-sites in β-Fe_2_O_3_ (designated Fe2, Fe3, Fe5, Fe6), and oxygen sites, respectively.

After releasing the pressure, both RO-Fe_2_O_3_ and PPV-Fe_2_O_3_ spontaneously reverted to the more thermodynamically stable α-Fe_2_O_3_ polymorph (see Fig. 2b). However, strikingly, the ζ-Fe_2_O_3_ phase retained its crystal structure after the pressure release, as shown in Fig. 2b,c. The presence of α-Fe_2_O_3_ and ζ-Fe_2_O_3_ phase in the sample after the pressure release was further evidenced by analyzing selective area electron diffraction (SAED) pattern (see Fig. 2e) where planes belonging to α-Fe_2_O_3_ and ζ-Fe_2_O_3_ phase were identified (other, not assigned planes most probably come from the matrix to which the sample was pressed). Moreover, the sample after pressure release features nanoparticles with an average particle size of 66.2 nm and log-normal particle size distribution from ~8 to ~150 nm (see Supplementary Figure S9), broadening significantly the diffraction lines in the XRD patterns. Then, the lattice constants of ζ-Fe_2_O_3_ at room temperature and atmospheric pressure were found to be *a* = 9.863 Å, *b* = 10.00 Å, *c* = 8.949 Å, *β* = 101.10°, and *V* = 850.4 Å^3^, and its crystal structure falls within the *I*2/*a* space group. The crystal structure of stable ζ-Fe_2_O_3_ is shown in Fig. 3 and Supplementary Figure S10; the atomic coordinates of the iron and oxygen sites in the ζ-Fe_2_O_3_ crystal structure are listed, together with lattice parameters, cell volume, and *R*_wp_-factor, in Table 2.

**Figure 4:**
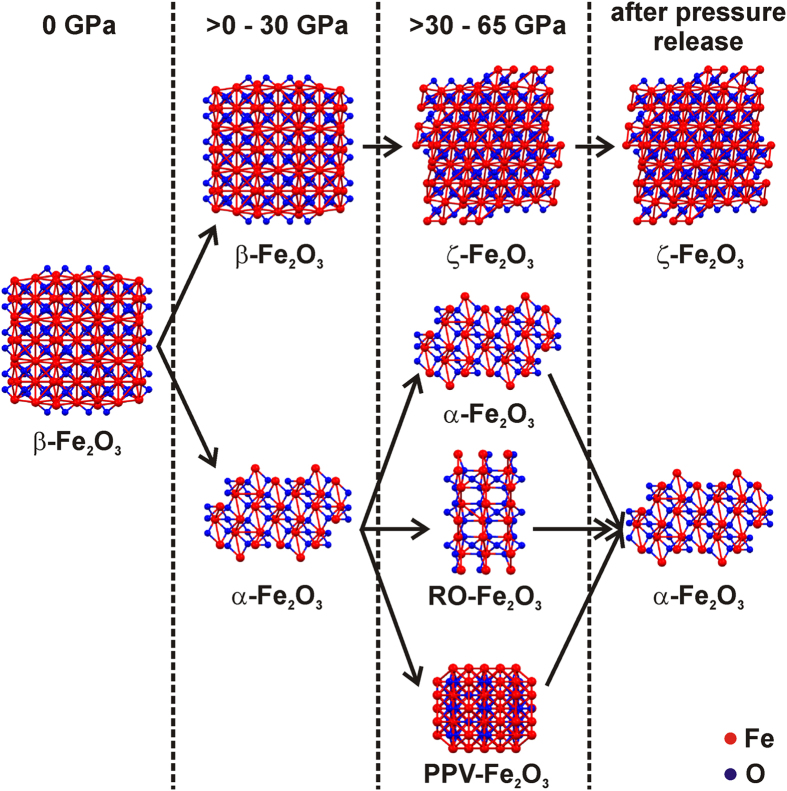
The proposed mechanism of b-Fe_2_O_3_ transformation under pressure.

No iron(III) oxide phase with a monoclinic crystal structure that is stable at atmospheric pressure and room temperature has ever been identified before. Thus, ζ-Fe_2_O_3_ can be regarded as a new member of the iron(III) oxide polymorphic family. It is known that the stability of nanoscale Fe_2_O_3_ polymorphs is governed by two factors: the Gibbs free energy of the different *i*-Fe_2_O_3_ phases (*i* = α, β, γ, ε), and the energy barrier associated with the phase transformation^25^. These two parameters in turn depend on many factors such as different phases’ kinetics of formation and (nano)structural properties of the phases’ particles such as their surface-to-volume ratios. The Gibbs free energy involves the chemical potential and the surface energy. It is generally accepted that surface energy and surface stress/strain are the key properties of nanoparticles that determine the formation and stability of crystalline phases. Because both parameters are strongly related to the nanoparticles’ dimensions, the extent to which a given applied pressure can modify the particles’ crystal structure depends on their size. Therefore, at high pressures, smaller β-Fe_2_O_3_ nanoparticles tend to transform into ζ-Fe_2_O_3_ while larger β-Fe_2_O_3_ nanoparticles primarily transform into α-Fe_2_O_3_ and then to perovskite and post-perovskite Fe_2_O_3_ phases (see Fig. 4). We hypothesize that the pressure treatment also affects the chemical potential of ζ-Fe_2_O_3_ and that this change (together with changes in the particles’ surface energy) causes the Gibbs free energy of the ζ-Fe_2_O_3_ phase to become lower than that of α-Fe_2_O_3_ and β-Fe_2_O_3_ over a wide range of pressures and temperatures. Consequently, it remains stable when the pressure is released. Additionally, pressure effect on nanoparticles may be considered as one of the reasons for the larger volume of ζ-Fe_2_O_3_ compared to β-Fe_2_O_3_ and for ζ-Fe_2_O_3_ to be maintained after pressure release. For example, in the case of CeO_2_ nanoparticles, volume expansion by pressure application has been reported due to the difference in the pressure-induced stress between the surface and the core of the nanoparticles^46^. Such nanosize effect may be contributing in the present system as well since β-Fe_2_O_3_ is obtained as nanoparticles. Furthermore, the remaining stress in the nanoparticles after pressure release may also explain the reason for ζ-Fe_2_O_3_ to remain under atmospheric pressure.

**Figure 5:**
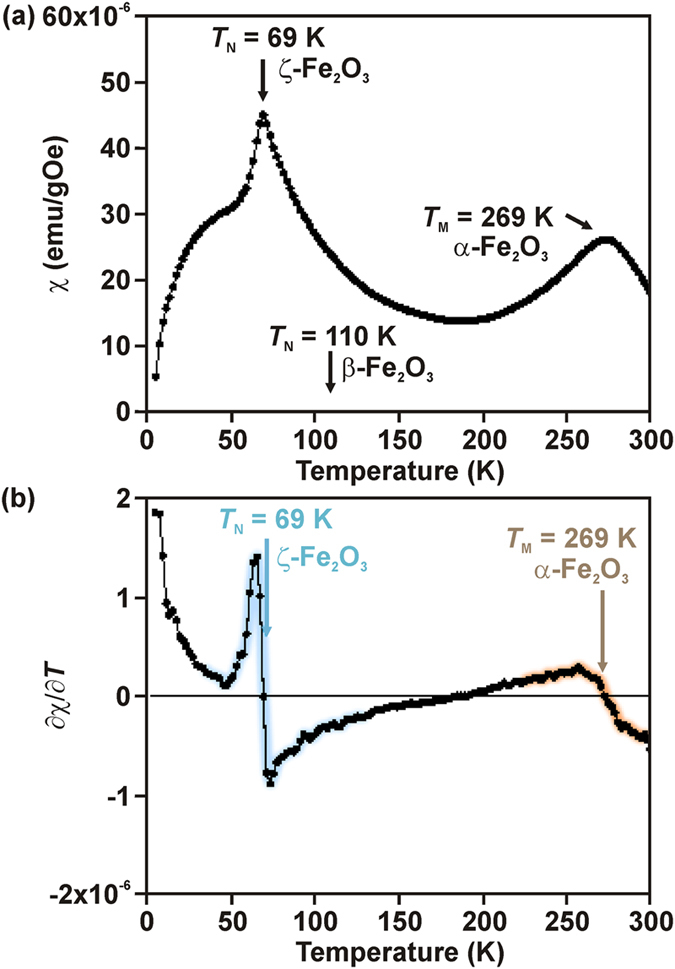
Magnetic property. Thermal evolution of the (**a**) magnetic susceptibility (*χ*) and (**b**) ∂*χ*/∂*T* of the sample after pressure release, monitored under an external magnetic field of 20 kOe (*T*_N_ marks the Néel temperature of ζ-Fe_2_O_3_ and *T*_M_ represents the Morin transition temperature of α-Fe_2_O_3_). Light blue and brown shades indicate the signals from ζ-Fe_2_O_3_ and α-Fe_2_O_3_, respectively.

The magnetic properties of the sample after the pressure release were investigated by measuring the temperature dependence of its mass susceptibility, *χ* (see Fig. 5a). Its *χ* profile contains two pronounced maxima, one at ~69 K (designated *T*_N_) and the other at ~269 K (designated *T*_M_). On moving away from these temperatures, *χ* decreases, indicating a transition to an antiferromagnetic state. The profile of the maximum at ~269 K resembles that of the Morin transition of α-Fe_2_O_3_, i.e. the transition from a weakly ferromagnetic regime to an antiferromagnetic state accompanied by a 90° spin reorientation (from the α-Fe_2_O_3_ basal plane to the *c*-axis direction). The second maximum is associated with a sharp peak and indicates the Néel temperature of the ζ-Fe_2_O_3_ phase. The transition at ~69 K shows features characteristic of a second-order thermodynamic transition, thus *T*_N_ can be regarded as the thermodynamic temperature of the passage from a magnetically disordered (paramagnetic) state to a magnetically ordered (antiferromagnetic) regime. The origin of the fall in the Néel temperature ζ-Fe_2_O_3_ with respect to β-Fe_2_O_3_ can be explained in terms of changes in the lattice volume. The lattice volume of ζ-Fe_2_O_3_ is larger than that of β-Fe_2_O_3_, resulting in the decrease in strength of superexchange interactions. Following the equation reported by Bloch^47^, ∂ln *J* = γ × ∂ln *V* (where *J* is the superexchange parameter (exchange integral), *V* is the lattice volume, and γ is the constant value given as –10/3), it turns out that the *J* value decreased as 93% by the phase transformation from β-Fe_2_O_3_ to ζ-Fe_2_O_3_. As *T*_N_ depends on *J*, the decrease in *J* is then macroscopically manifested in the decrease in *T*_N_ as experimentally evidenced from the temperature behavior of *χ*. Here, it should be stressed that the decrease in *T*_N_ does not result from strains or defects because the temperature differential of *χ* (i.e., ∂*χ*/∂*T*) at *T*_N_ (69 K) is sharper than that at 269 K belonging to *T*_M_ of α-Fe_2_O_3_ (see Fig. 5b). If the decrease in *T*_N_ in β-Fe_2_O_3_ was promoted by the local disorder, the peak at *T*_N_ should be broadened. Therefore, *T*_N_ at 69 K is considered as the magnetic transition temperature of the ζ-Fe_2_O_3_ phase.

## Conclusions

The pressure-induced transformation of the rare β-Fe_2_O_3_ phase has been studied for the first time, leading to the identification of a new iron(III) oxide polymorph, ζ-Fe_2_O_3_. The transformation of β-Fe_2_O_3_ into ζ-Fe_2_O_3_ occurs above 30 GPa and the new phase withstands pressures of up to ~70 GPa, which is well above the thresholds for the pressure-induced transformations of α-Fe_2_O_3_ or γ-Fe_2_O_3_. More strikingly, ζ-Fe_2_O_3_ remains stable after pressure release and at room temperature. This remarkable observation is explained in terms of its Gibbs free energy (and surface energy), which is partly due to structural properties inherited from its precursor material (small β-Fe_2_O_3_ nanoparticles) and partly due to stabilizing structural changes that occur during high pressure treatment. Its stability is thus strongly linked to the nanoscale dimensions of its particles. It has a monoclinic crystal structure belonging to the *I*2/*a* space group (*a* = 9.683 Å, *b* = 10.00 Å, *c* = 8.949 Å, *β* = 101.10°, and *V* = 850.4 Å^3^). The ζ-Fe_2_O_3_ phase behaves in an antiferromagnetic manner with a Néel transition temperature of ~69 K. It may also have other interesting electronic, optical, and transport properties that would lend themselves to practical applications. Thus, in future, two challenges are viewed to be of significance importance stimulating further research in the iron(III) oxide realm: (i) to develop new methods for preparing ζ-Fe_2_O_3_ from ultrafine β-Fe_2_O_3_ nanoparticles, possibly by exploiting spatial restrictions, controlling the level of interparticle interactions (aggregation) during transformation, and using thermal rather than pressure treatment; and (ii) to study the pressure-induced transformations of rare ε-Fe_2_O_3_.

## Methods

### Synthesis of b-Fe_2_O_3_ nanoparticles

β-Fe_2_O_3_ nanoparticles were synthesized by the thermally-induced solid-state reaction of NaCl with Fe_2_(SO_4_)_3_ in air followed by post-processing separation based on dissolution of all by-products in water as described previously^45^.

### *In-situ* high-pressure X-ray diffraction experiments with synchrotron radiation

High-pressure X-ray powder diffraction experiments with synchrotron radiation were performed using a diamond anvil cell high-pressure apparatus^48^. A powdered β-Fe_2_O_3_ sample was loaded into a 50–100 μm diameter hole that was drilled into a rhenium gasket. Several ruby crystals were also put into the sample chamber. No pressure transmitting medium was used in this study. The applied pressure was determined by monitoring the fluorescence line of ruby^49^ and ranged from 0.1 MPa to 64.4 GPa. In all cases, the desired pressure was established by gradually increasing the applied pressure. At selected pressures, the sample was probed by angle-dispersive X-ray diffraction using the NE1A synchrotron beam line at the Photon Factory in Japan. A monochromatic incident X-ray beam with a wavelength of *λ* ≈ 0.41 Å was used. The X-ray beams were collimated to a diameter of 30 μm, and the angle-dispersive X-ray diffraction patterns were obtained on an imaging plate (Rigaku) with 3000 × 3000 pixels. The distance between the sample and the detector was ~320 mm. The observed intensities on the imaging plates were integrated as a function of 2*θ* in order to obtain conventional one-dimensional diffraction profiles; details of the experimental procedure are presented elsewhere^50^.

For indexing the peaks and searching the space group, the JADE software from Materials Data., Inc., (MDI) was employed. Rietveld analyses were performed using the PDXL Integrated X-ray powder diffraction software package from Rigaku. When analyzing the synchrotron radiation XRD patterns, the phase fractions, lattice parameters, peak width, and decay parameters were refined.

### Conventional experimental techniques – X-ray powder diffraction, ^57^Fe Mössbauer spectroscopy, magnetization measurements, and transmission electron microscopy

XRD analysis of the initial β-Fe_2_O_3_ sample was recorded on a PANalytical X´Pert PRO diffractometer in the Bragg-Brentano geometry, equipped with an iron-filtered CoK_α_ radiation source, an X´Celerator detector, a programmable divergence and diffracted beam anti-scatter slits. Generally, 200 μL of a sample suspension was dropped onto a zero–background single–crystal Si slide, allowed to dry under vacuum at room temperature and scanned in continuous mode (resolution of 0.017° in 2*θ*, scan speed of 0.008° in 2*θ* per second, 2*θ* range from 20° to 105°) under ambient conditions. The commercially available standards SRM640 (Si) and SRM660 (LaB6) supplied by the National Institute of Standards and Technology (NIST) were used to evaluate line positions and instrumental line broadening, respectively. The acquired pattern was processed using the X´Pert HighScore Plus software package (PANalytical, The Netherlands) in combination with the PDF-4+ and ICSD databases.

The room-temperature transmission ^57^Fe Mössbauer spectrum of the initial β-Fe_2_O_3_ sample was recorded with a Mössbauer spectrometer operating in constant acceleration mode and equipped with a 50 mCi ^57^Co(Rh) source of γ-rays. The collected Mössbauer spectrum was fitted using Lorentzian line shapes with the MossWinn software package based on the least-square method. The isomer shift values were referenced to a metallic α-Fe sample at room temperature.

TEM images and SAED pattern were obtained using a JEOL JEM–2010 electron microscope operating at 160 kV with a point–to–point resolution of 1.9 Å. For each measurement, a drop of a very dilute dispersion of the sample was placed on a copper grid with a holey carbon film and allowed to dry under vacuum at room temperature. HRTEM images were obtained using a TITAN 60–300 high-resolution transmission electron microscope with an X-FEG type emission gun, operating at 80 kV. For HRTEM analyses, the powder β-Fe_2_O_3_ sample was dispersed in ethanol and ultrasonicated for 5 minutes. One drop of the resulting suspension was then placed on a copper grid covered with a holey carbon film and allowed to dry at room temperature.

A superconducting quantum interference device (SQUID) magnetometer (MPMS XL-7 type, Quantum Design, U.S.A.) was used to measure the magnetization of the β-Fe_2_O_3_ sample after pressure release. The temperature evolution of the sample magnetization was recorded under an external magnetic field of 20 kOe in the sweep mode at temperatures ranging from 5 to 300 K. The gathered data were corrected to account for the paramagnetic and diamagnetic contributions from the material the sample was pressed into.

## Additional Information

**How to cite this article**: Tuček, J. *et al.* Zeta-Fe_2_O_3_ - A new stable polymorph in iron(III) oxide family. *Sci. Rep.* **5**, 15091; doi: 10.1038/srep15091 (2015).

## Supplementary Material

Supplementary Information

## Figures and Tables

**Table 1:** Values of the Mössbauer hyperfine parameters, derived from the fitting of the recorded room-temperature ^57^Fe Mössbauer spectrum of the initial b-Fe_2_O_3_ sample, where *δ* is the isomer shift, D*E*_*Q*_ is the quadrupole splitting, *B*_hf_ is the hyperfine magnetic field, and RA is the relative spectral area of individual spectral components.

Sample	Component	*δ *± 0.01(mm/s)	D*E*_*Q*_* *± 0.01(mm/s)	*B*_hf_* *± 0.3(T)	RA* *± 1(%)	Assignment
β-Fe_2_O_3_	Doublet	0.36	0.92	—	71	β-Fe_2_O_3_ – b-sites
	Doublet	0.37	0.71	—	23	β-Fe_2_O_3_ – d-sites
	Sextet	0.36	−0.20	52.0	6	α-Fe_2_O_3_

**Table 2:** Crystal structure and lattice parameters of z-Fe_2_O_3_ phase at atmospheric pressure and room temperature with atomic coordination of iron and oxygen atoms.

Crystal system			Monoclinic
Space group			*I*2/*a* (No. 15)
*a* (Å)			9.683(15)
*b* (Å)			10.00(2)
*c* (Å)			8.949(12)
*β* (°)			101.10(6)
*V* (Å^3^)			850.4(2)
*Z*			16
*R*_wp_ (%)			0.42
	*x*/*a*	*y*/*b*	*z*/*c*
Fe(1)	0.000	0.000	0.000
Fe(2)	0.250	0.296	0.000
Fe(3)	0.750	0.225	0.000
Fe(4)	0.000	0.500	0.000
Fe(5)	0.216	0.000	0.750
Fe(6)	0.500	0.250	0.716
O(1)	0.083	0.371	0.147
O(2)	0.417	0.629	0.647
O(3)	0.147	0.083	0.371
O(4)	0.353	0.917	0.871
O(5)	0.371	0.147	0.083
O(6)	0.871	0.353	0.917
