# Supplementary material for: Zeta-Fe2O3 – A new stable polymorph in iron(III) oxide family
Source: Sci Rep. 2015 Oct 15;5:15091. doi: 10.1038/srep15091 (PMC4606832; doi:10.1038/srep15091)
Supplement: Supplementary Information [file srep15091-s1.pdf]

SUPPLEMENTARY INFORMATION

Zeta-Fe_2O_3 – A new stable polymorph in iron(III) oxide family

Jiří Tuček,[1] Libor Machala,[1] Shigeaki Ono,[2] Asuka Namai,[3] Marie Yoshikiyo,[3] Kenta Imoto,[3] Hiroko Tokoro,[3] Shin-ichi Ohkoshi,[3]* and Radek Zbořil[1,]*

[1] *Regional Centre of Advanced Technologies and Materials, Departments of Physical Chemistry and Experimental Physics, Faculty of Science, Palacky University, Slechtitelu 11, 783 71 Olomouc, Czech Republic.*

[2] *Research and Development Center for Ocean Drilling Science, Japan Agency for Marine-Earth Science and Technology, 2-15 Natsushima-cho, Yokosuka-shi, Kanagawa 237-0061, Japan.*

[3] *Department of Chemistry, School of Science, The University of Tokyo, 7-3-1 Hongo, Bunkyo-ku, Tokyo 113-0033, Japan.*

* Authors to whom correspondence should be addressed: Phone: +420 585634337, Fax: +420 585634761, E-mail address: radek.zboril@upol.cz (Radek Zbořil); Phone: +81-3-5841-4331, Fax: +81-3-3812-1896, E-mail address: ohkoshi@chem.s.u-tokyo.ac.jp (Shin-ichi Ohkoshi)

Number of pages: 11

Number of figures:10

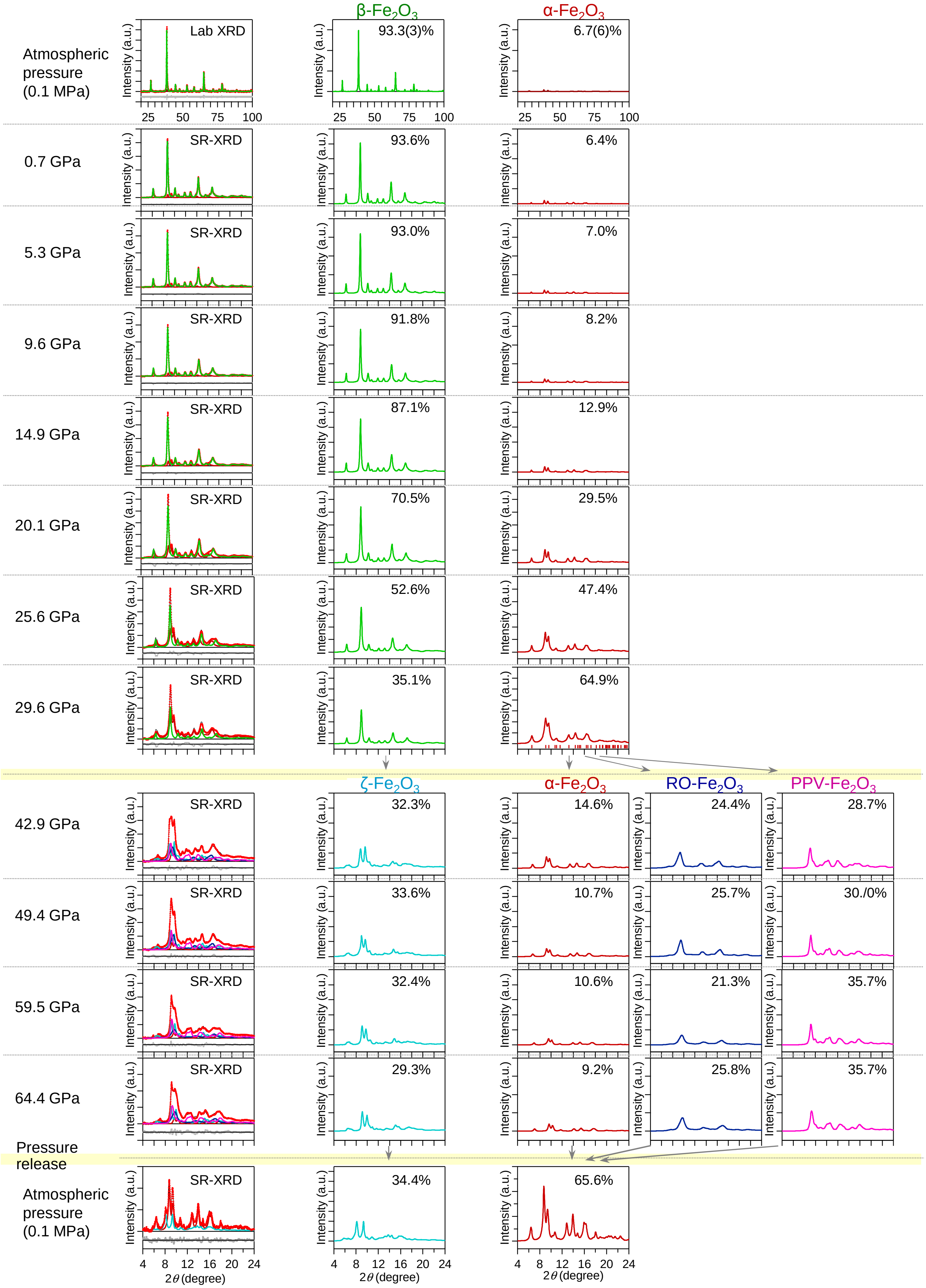

Supplementary Figure S1. Left column shows the SR-XRD patterns before applying pressure and in the pressure-increasing process at 0.7–64.4 GPa, and at 0.1 MPa, atmospheric pressure, after pressure release. Red dots, black lines, and gray dots are the observed patterns, fitted patterns by Rietveld analyses, and their differences, respectively. Right columns show the fitted patterns for each phase.

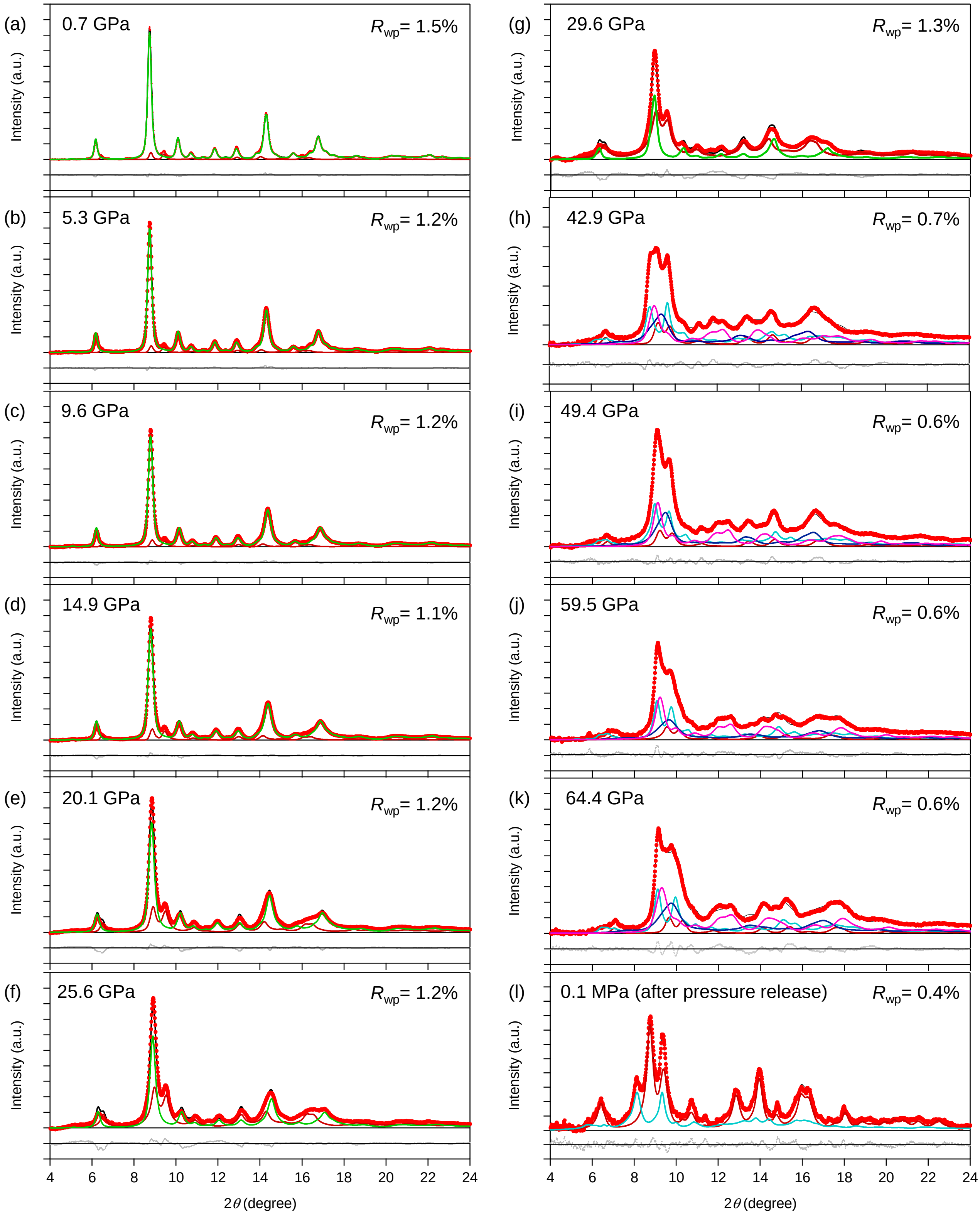

Supplementary Figure S2. SR-XRD patterns (a–k) in the pressure-increasing process at 0.7–64.4 GPa, and (l) at 0.1 MPa, atmospheric pressure, after pressure release. Red dots, black lines, and gray dots are the observed patterns, fitted patterns by Rietveld analyses, and their differences, respectively. Fitted patterns for each phase are shown with green lines (β-Fe_2O_3), brown lines (α-Fe_2O_3), navy blue lines (RO-Fe_2O_3), pink lines (PPV-Fe_2O_3), and light blue lines (ζ-Fe_2O_3).

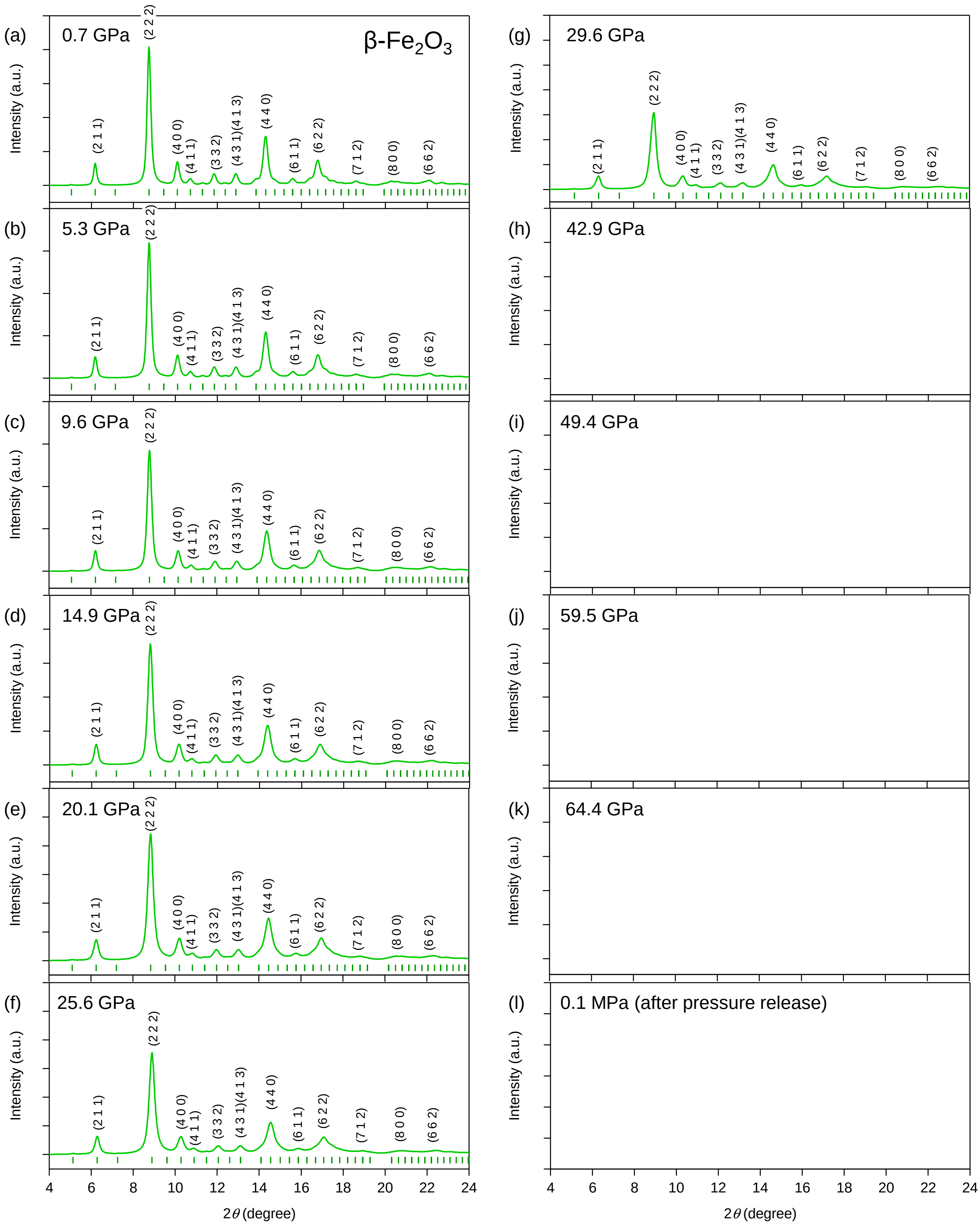

Supplementary Figure S3. Green lines are the fitted SR-XRD patterns of β-Fe_2O_3 in the pressure-increasing process at 0.7–64.4 GPa (a–k), and at 0.1 MPa, atmospheric pressure, after pressure release (l) obtained by Rietveld analyses. β-Fe_2O_3 is observed from 0.7 GPa to 29.6 GPa. Green tick marks indicate the calculated positions of the Bragg reflections. Panels (h) to (l) are empty as β-Fe_2O_3 does not exist at pressures from 42.9 to 64.4 GPa and is not present in the sample after pressure release.

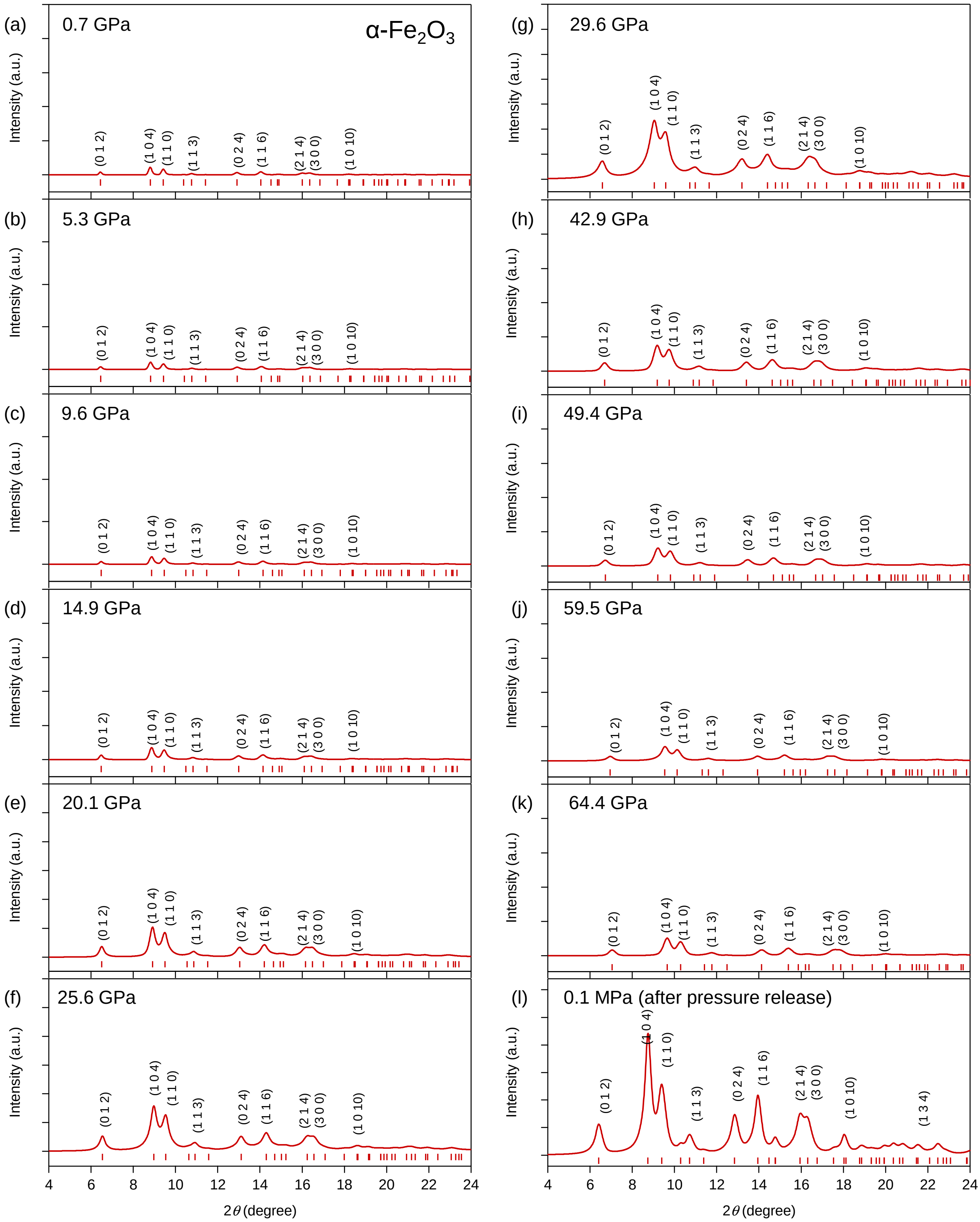

Supplementary Figure S4. Brown lines are the fitted SR-XRD patterns of α-Fe_2O_3 in the pressure-increasing process at 0.7–64.4 GPa (a–k), and at 0.1 MPa, atmospheric pressure, after pressure release (l) obtained by Rietveld analyses. α-Fe_2O_3 is observed at all pressures. Brown tick marks indicate the calculated positions of the Bragg reflections.

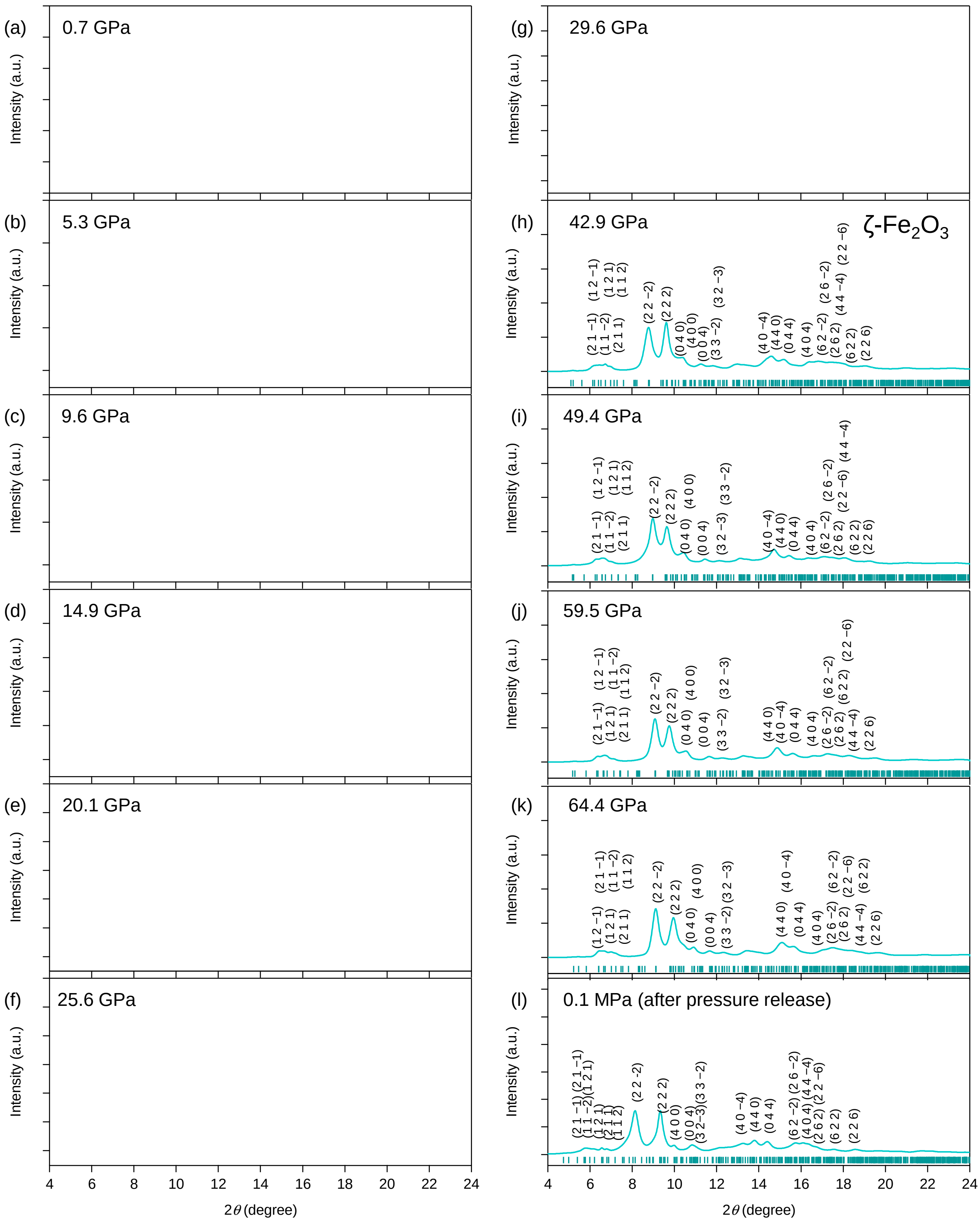

Supplementary Figure S5. Light blue lines are the fitted SR-XRD patterns of ζ-Fe_2O_3 in the pressure-increasing process at 0.7–64.4 GPa (a–k), and at 0.1 MPa, atmospheric pressure, after pressure release (l) obtained by Rietveld analyses. ζ-Fe_2O_3 is observed above 40 GPa and after pressure release. Light blue tick marks indicate the calculated positions of the Bragg reflections. Panels (a) to (g) are empty as ζ-Fe_2O_3 does not exist at pressures below 42.9 GPa.

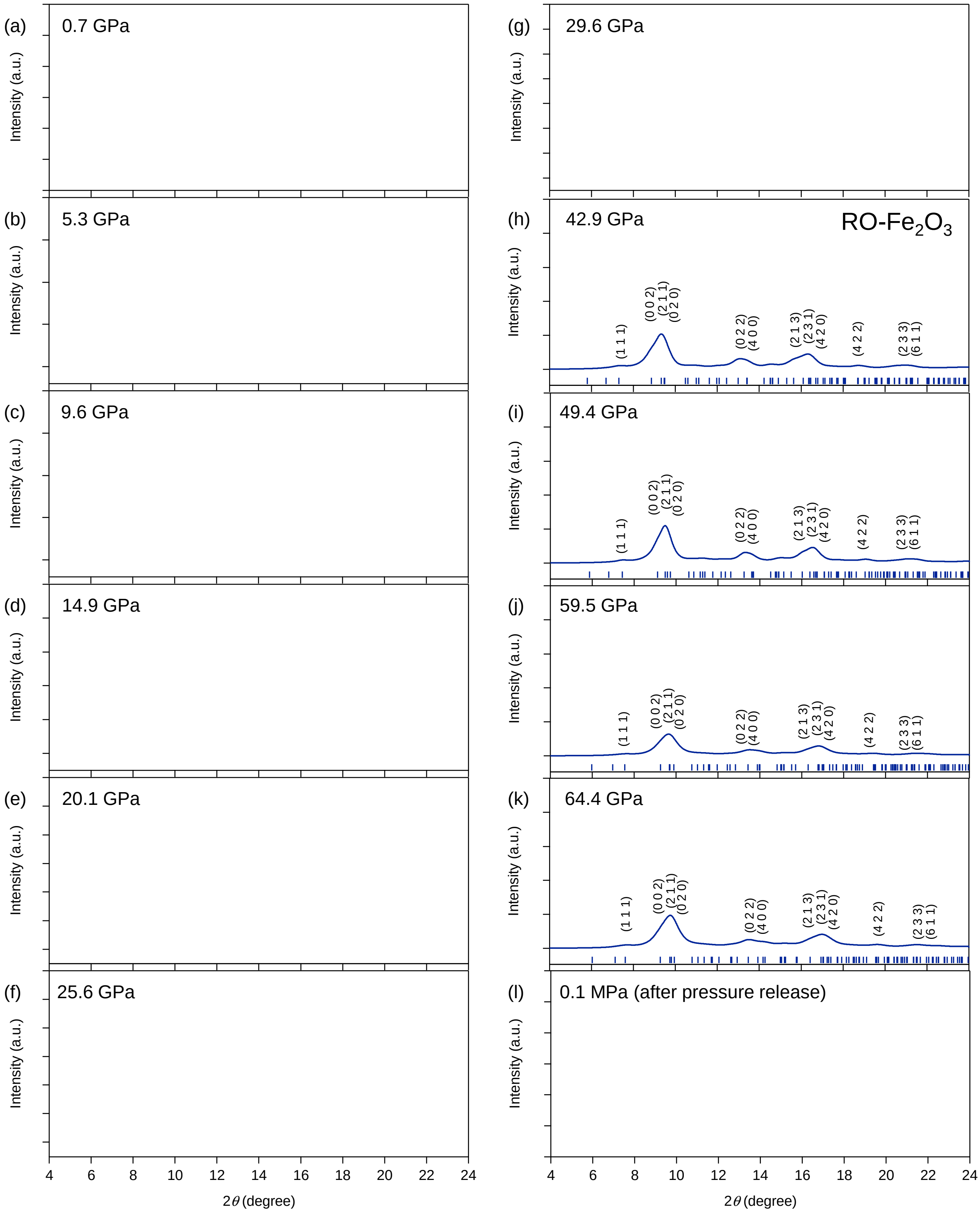

Supplementary Figure S6. Navy blue lines are the fitted SR-XRD patterns of RO-Fe_2O_3 in the pressure-increasing process at 0.7–64.4 GPa (a–k), and at 0.1 MPa, atmospheric pressure, after pressure release (l) obtained by Rietveld analyses. RO-Fe_2O_3 is observed above 40 GPa. Navy blue tick marks indicate the calculated positions of the Bragg reflections. Panels (a) to (g) and panel (l) are empty as RO-Fe_2O_3 does not exist at pressures below 42.9 GPa and after pressure release.

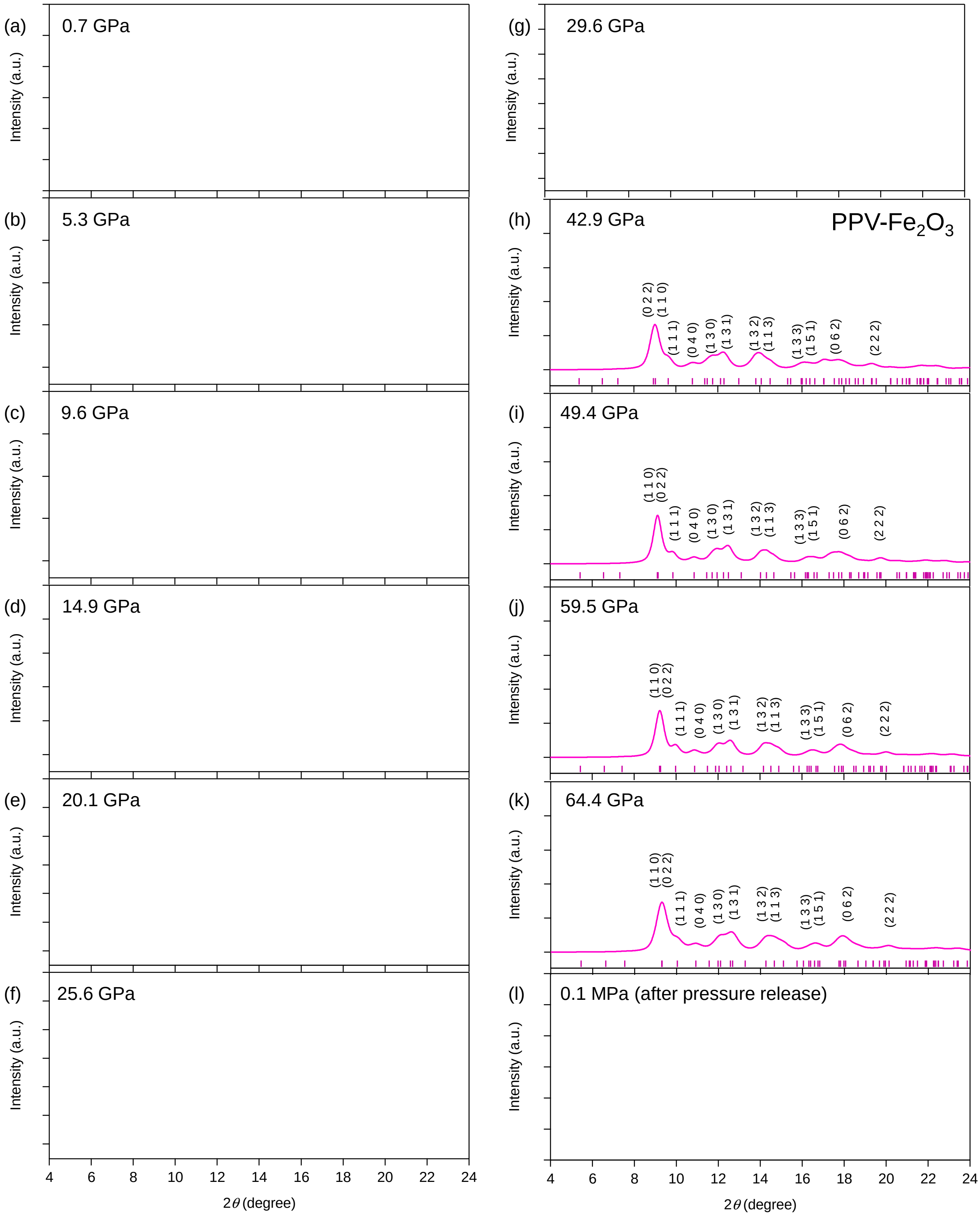

Supplementary Figure S7. Pink lines are the fitted SR-XRD patterns of PPV-Fe_2O_3 in the pressure-increasing process at 0.7–64.4 GPa (a–k), and at 0.1 MPa, atmospheric pressure, after pressure release (l) obtained by Rietveld analyses. PPV-Fe_2O_3 is observed above 40 GPa. Pink tick marks indicate the calculated positions of the Bragg reflections. Panels (a) to (g) and panel (l) are empty as PPV-Fe_2O_3 does not exist at pressures below 42.9 GPa and after pressure release.

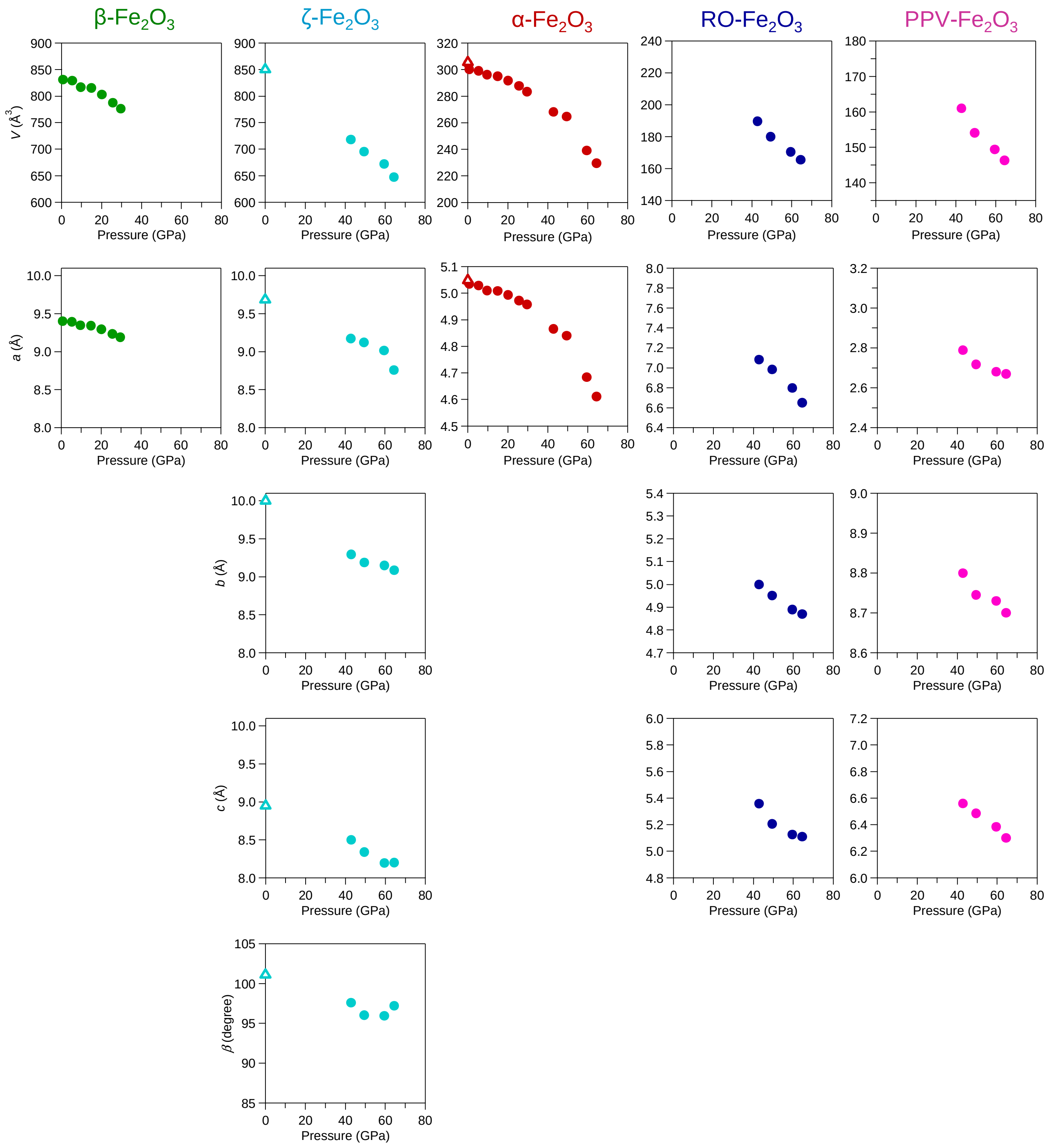

Supplementary Figure S8. Pressure dependence of cell volume and lattice parameters of β-Fe_2O_3 (*V*, *a*), ζ-Fe_2O_3 (*V*, *a*, *b*, *c*, *β*), α-Fe_2O_3 (*V*, *a*, *c*), RO-Fe_2O_3 (*V*, *a*, *b*, *c*), and PPV-Fe_2O_3 (*V*, *a*, *b*, *c*).

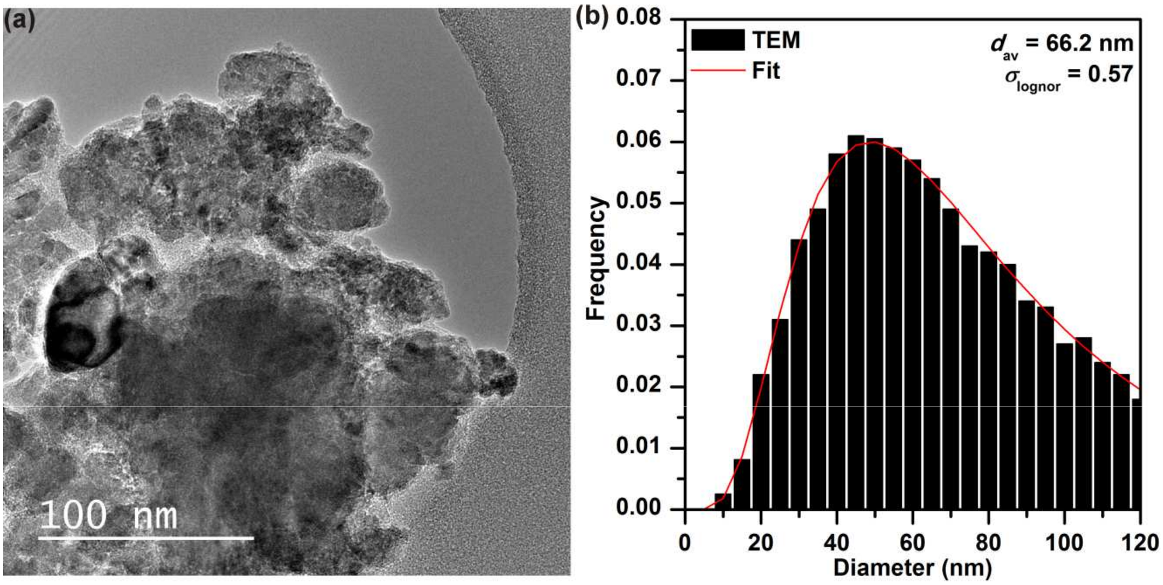

Supplementary Figure S9. (a) Representative HRTEM image of the sample after pressure release and (b) particle size distribution derived from analysis of HRTEM images. The experimental distribution profile can be well fitted with the log-normal distribution curve with an average particle size of 66.2 nm and a log-normal standard deviation of 0.57.

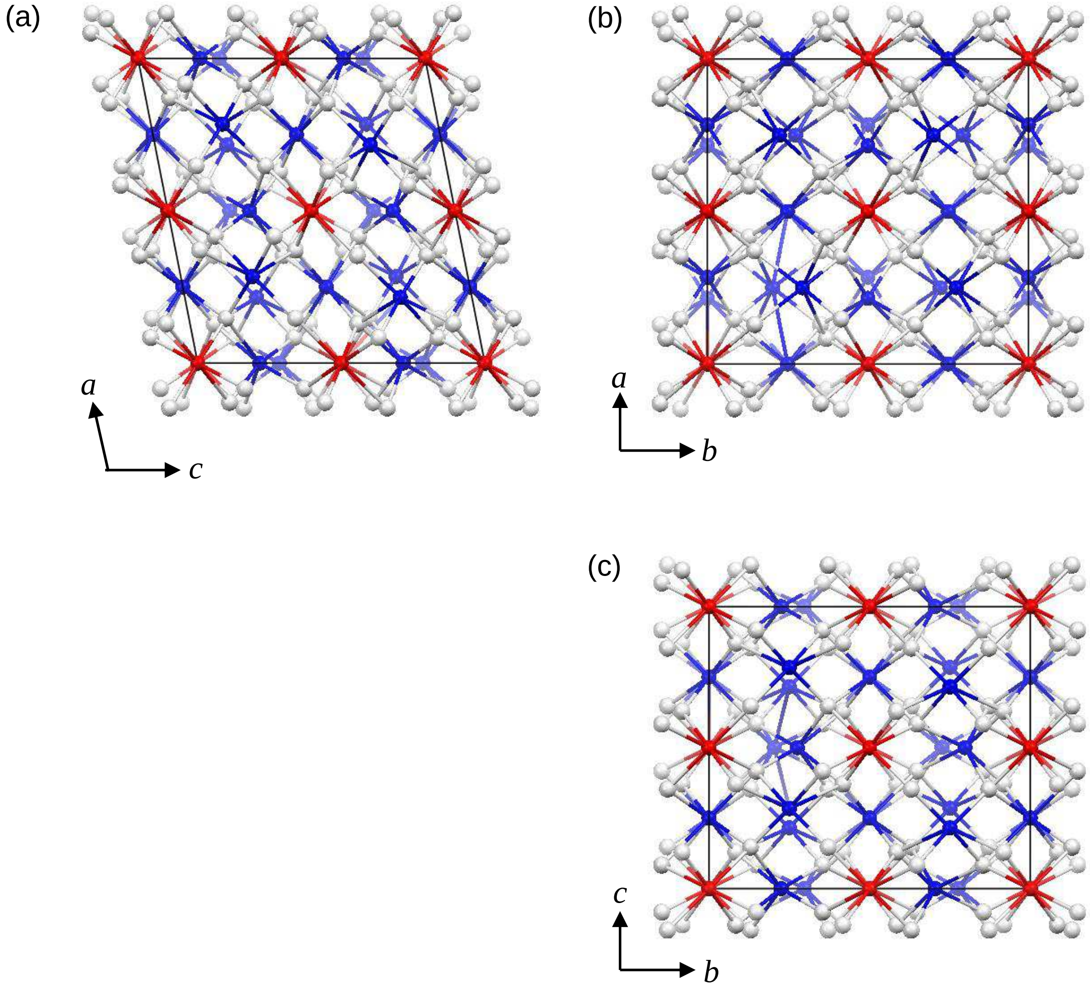

Supplementary Figure S10. Crystal structure of ζ-Fe_2O_3 after pressure release viewed from (a) *a*-axis, (b) *b*-axis, and (c) *c*-axis. The size of the atoms indicate the thermal factors obtained from Rietveld analysis.
